# Essential and dispensable domains of DivIVA for walled growth in filamentous Actinomycetota

**DOI:** 10.1098/rsob.250213

**Published:** 2025-11-05

**Authors:** Maarten Lubbers, Belmin Bajramović, Véronique Ongenae, Joost Willemse, Dieuwertje de Bruin, Niels Mulder, Le Zhang, Bastienne Vriesendorp, Francisco Barona-Gomez, Ariane Briegel, Gilles P. van Wezel, Klas Flärdh, Dennis Claessen

**Affiliations:** ^1^ Leiden University Institute of Biology, Leiden, Zuid-Holland, The Netherlands; ^2^ Integrative Structural Cell Biology Unit, Institut Pasteur, Paris, Île-de-France, France; ^3^ Netherlands Institute of Ecology (NIOO-KNAW), Wageningen, Gelderland, The Netherlands; ^4^ Lund University, Lund, Skåne County, Sweden

**Keywords:** hyphal growth, Streptomycetaceae, L-forms, cell wall deficiency, morphogenesis

## Abstract

The morphogenetic protein DivIVA exhibits diverse functions across bacterial phyla. In Bacillota, DivIVA is primarily involved in cell division, whereas in Actinomycetota, it plays a central role in coordinating polar growth. Due to its essentiality in Actinomycetota, gaining insight into its structural functions is challenging. We studied truncated DivIVA proteins using a unique *divIVA* deletion mutant in cell wall-deficient *Kitasatospora viridifaciens* L-forms. DivIVA comprises an N-terminal domain consisting of a coiled-coil segment bearing a membrane-targeting structure at the N-terminal end, followed by an intercoil region, a larger coiled-coil and a C-terminal domain. Deleting either the intercoil or C-terminal region affected branching. We also created a minimized variant in which both were deleted simultaneously, retaining the N-terminal domain and the second coiled-coil. Expression of this variant caused severe growth defects. Cells showed increased hyphal width, thicker cell walls and frequent tip bursting. Finally, we successfully introduced chimeric DivIVA from the unicellular actinobacterium *Mycolicibacterium smegmatis* with the membrane targeting domain of *K. viridifaciens* DivIVA, demonstrating functional conservation within the phylum. By contrast, chimeric DivIVA proteins from *Bacillus subtilis* could not support growth, underscoring that polar growth is encoded in Actinomycetota-specific amino acid motifs in the first and second coiled-coils. These findings enhance our understanding of the structure–function relationship for DivIVA and present new opportunities to study polar growth.

**Figure:**
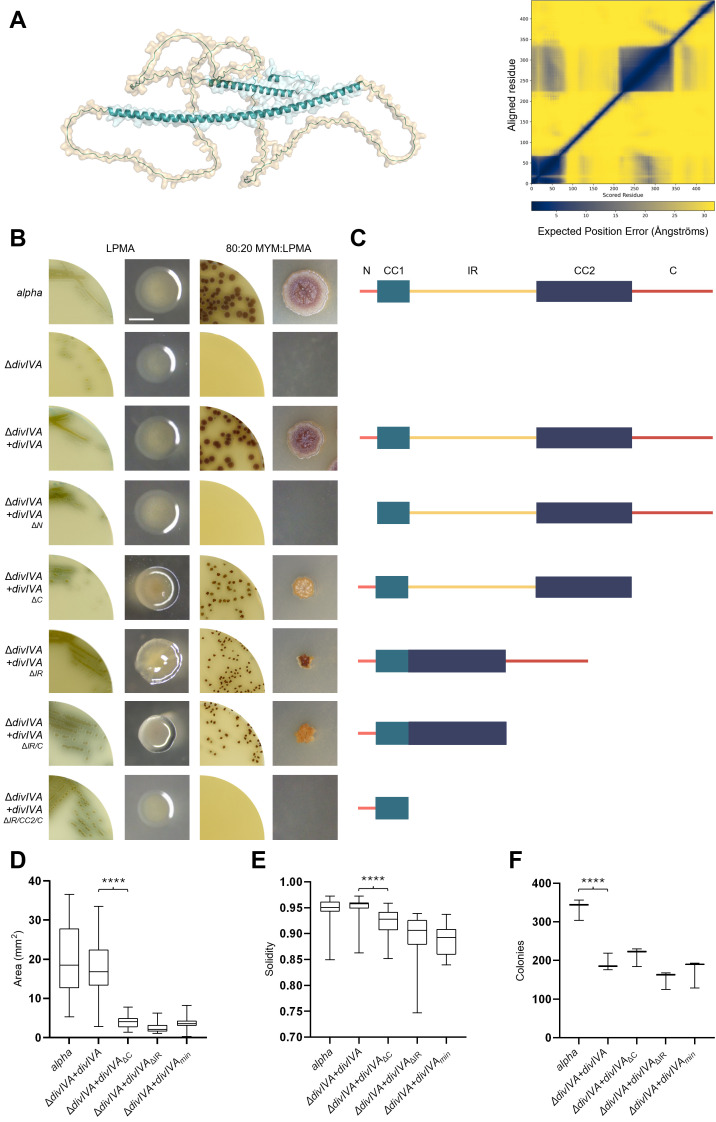

## 1. Introduction

Streptomycetaceae are filamentous bacteria that form multicellular networks. Unlike well-studied bacteria such as *Escherichia coli*, which grow by adding new cell material along their lateral walls [1], *Kitasatospora* and *Streptomyces* form so-called hyphae that grow by extension at their tips. During this process, known as polar growth, new material is added only at the ends of the filaments [2]. These filaments can also branch, leading to the formation of a network of interconnected hyphae, known as a mycelium. Polar growth and the formation of branches are orchestrated by DivIVA [2], a membrane-binding protein found at growing tips [3]. DivIVA is an essential protein, and partially depleting DivIVA results in the formation of abnormally shaped hyphae and irregular branching [4,5]. By contrast, overexpressing DivIVA causes peptidoglycan to be added at multiple new sites, triggering excessive lateral branching [3,5]. In other unicellular Actinomycetota (or Actinobacteria), such as *Mycolicibacterium smegmatis* and *Corynebacterium glutamicum*, DivIVA is also essential, where it plays a central role in coordinating polar growth [6–8].

In Streptomycetaceae, DivIVA consists of four domains: an N-terminal domain consisting of a coiled-coil segment bearing an N-terminal membrane-targeting sequence, followed sequentially by an intercoil region, a larger coiled-coil, and a C-terminal domain [5]. The N-terminal domain and the second coiled-coil region are highly conserved among Streptomycetaceae, in contrast to the variable intercoil region and the C-terminal domain [5]. Computer simulations predicted that the N-terminal domain binds to the membrane, with its affinity dependent on membrane lipid composition [9]. Furthermore, DivIVA forms a multimeric complex in streptomycetes [4]. In non-polarly growing bacteria, such as Bacillota (or Firmicutes), DivIVA is non-essential, while still playing a role in cell division. In *Bacillus subtilis*, the DivIVA protein targets the division septum and cell poles and contributes to the control of the topological specificity of cell division [10]. DivIVA also plays a role in sporulating cells, interacting with the chromosome segregation machinery to aid in positioning the *oriC* region at the cell pole [11]. Structural models based on crystallography indicate that the N-terminal membrane-binding domain of *B. subtilis* DivIVA forms a dimer with crossed-over loops, likely interacting with the membrane surface [12]. Further, models based on the crystal structures suggest that the C-terminal coiled-coil region is involved in oligomerization and forms a tetramer [12]. *B. subtilis* DivIVA associates with negatively curved membranes, possibly through molecular bridging of the curvature by DivIVA multimers [13].

A full understanding of the roles and mechanisms of DivIVA in Actinomycetota requires the ability to delete and functionally analyse this gene. This is made possible by using L-forms, which are bacterial cells that can proliferate without a cell wall and cell division machinery [14]. L-form proliferation is governed by biophysical principles, driven by increased membrane synthesis, leading to an imbalance in the cell’s surface-to-volume ratio [15,16]. Previously, an L-form strain of *Kitasatospora viridifaciens* was generated under laboratory conditions and named *alpha* [17]. *Alpha* only proliferates in its cell wall-deficient state when grown in osmoprotective media. When grown on medium with lower levels of osmolytes, *alpha* reverts to walled growth and forms mycelial networks comparable with the parental strain [17]. *Alpha* cannot sporulate since it is missing a 1.5 Mb segment of the right chromosomal arm [18]. Strain Δ*divIVA* was generated by deleting the *divIVA* gene, disabling the strain from reverting to walled, filamentous growth. Reintroduction of the native *divIVA* gene fully restored walled growth, showing efficient complementation *in trans* [19]. Using this system, we investigated which domains of DivIVA are critical for its function in steering walled growth in Streptomycetaceae. We created a functional minimized DivIVA variant comprising the N-terminal domain and the second coiled-coil. Removing both the intercoil region and the C-terminal domain led to a very strong phenotype, with increasing cross-wall formation, highly irregular hyphal diameter, increased cell-wall thickness and frequent lytic events. Furthermore, this minimized DivIVA protein structurally resembles DivIVA from unicellular bacteria, giving us a better understanding of structure–function relationships. This approach establishes a powerful platform for dissecting the functional architecture of essential morphogenetic proteins and enables novel strategies for engineering cell shape and growth in filamentous Actinomycetota.

## 2. Material and methods

### 2.1. Strains and media

Bacterial strains used in this study are listed in electronic supplementary material, table S1. *Escherichia coli* was grown at 30°C or 37°C at 200 RPM in LB. *Kitasatospora viridifaciens* DSM40239 was grown on solid MYM medium for 3 days [20] or as a liquid-shaken culture in TSBS (tryptone soy broth with 10% sucrose) at 200 RPM [21]. L-form strains were grown on LPMA plates supplemented with 5% (v/v) horse serum and 25 mM MgCl_2_ [17], whereas L-phase broth (LPB) [17] was used for growth in liquid (at 100 RPM). To facilitate the reversion of the Δ*divIVA* mutant carrying different *divIVA* plasmids, strains were first grown in LPB medium for 2 days at 30°C at 100 RPM until reaching an OD of 0.5. 200 µl of these cultures were inoculated on reversion plates (MYM:LPMA (4:1)) plates and grown at 30°C for 3 days [22].

### 2.2. Plasmid construction and transformation

Plasmids used in this study are listed in electronic supplementary material, table S2. All primers are listed in electronic supplementary material, table S3. To isolate genomic DNA of *K. viridifaciens*, the protocol was performed as described by Kieser *et al.* [21]. Fragments were amplified using Q5 DNA polymerase (New English Biolabs). For Gibson assembly, pIJ82 containing the *gapAp* promoter [23] was used as the backbone vector following digestion with *Xba*I and *Nde*I. Gibson-assembled products were transformed into competent *E. coli* TOP10 cells. Plasmids were isolated using the Nucleospin plasmid Easypure kit (BIOKÉ) and confirmed by Sanger sequencing (Macrogen). Codon-optimized *divIVA* genes from *Bacillus subtilis* and *Mycolicibacterium smegmatis* were synthesized by Integrated DNA Technologies and Twist Bioscience, respectively. Plasmid transformation into the *K. viridifaciens* Δ*divIVA* mutant was carried out following the protocol described by Zhang *et al.* [19].

### 2.3. Microscopy and imaging

#### 2.3.1. Light microscopy

Microscopy analysis of mycelia grown in liquid was conducted using a Zeiss Axio Lab A1 upright Microscope, equipped with an Axiocam MRc (Zeiss) using a 40× magnification. Plate images were made using the Perfection V600 scanner (Epson). Colony photos were made using a Mikrocam SP 5.0 microscope camera (Bresser) connected to a SteREO Discovery V8 stereomicroscope (Zeiss).

To visualize growth over time, metal growth chambers shaped like a microscope slide with a 13 mm hole in the centre were used. In these chambers, filtered MYM medium solidified with 1% agarose was enclosed between a Lumox Biofoil 25 membrane (Greiner Bio-One) and a cover slip [3]. Mycelia were imaged at 30°C under a Axio Observer.Z1 inverted light microscope (Zeiss), using a Plan-Apochromat 40×/1.4 Ph2 objective and an ORCA Flash 4.0 LT camera (Hamamatsu).

To observe the bursting of hyphae in detail, liquid culture was spun down for 3 min at 0.4 rcf. To concentrate cells, the supernatant containing smaller mycelia was transferred to a new tube and spun down for 10 min at 13.000 rpm and resuspended 1:50 in filtered TSBS. A CellASIC B04A-03 microfluidic plate (Merck) was rinsed and primed with filtered TSBS. Further steps were followed as described by Passot *et al.* [24]. An inverted Axio Observer.Z1 (Zeiss) was used with a 100× phase contrast objective.

#### 2.3.2. Fluorescence microscopy

For DivIVA::eGFP localization studies, cells were imaged under an Axio Observer.Z1 inverted light microscope, using a Plan-Apochromat 100×/1.4 Oil Ph3 objective and an ORCA Flash 4.0 LT camera (Hamamatsu). L-form cells were placed on 1% agarose in P-buffer with 0.5 μg ml^−1^ FM4-64. Metal growth chambers shaped as a microscope slide with a 13 mm hole in the centre were used, where the medium was enclosed between a Lumox Biofoil 25 membrane (Greiner Bio-One) and a cover slip [3]. Z-stacks of images were acquired, with 25 planes with a 270 nm distance. To enable image deconvolution, point-spread functions (PSF) were determined using beads with excitation/emission wavelengths of 505/515 nm (green) and 633/660 nm (deep red) from the PS-Speck Microscope Point Source Kit (Invitrogen). The beads were imaged and PSFs calculated using the Deconvolution module in Zen software (Zeiss, ver 3.9). Image stacks were captured with Z-spacing as above, and deconvolution was applied to the whole stack using the constrained iterative algorithm of Zen and the determined PSFs. Orthogonal projections were made from four focal planes through the centre of the cells.

#### 2.3.3. Scanning electron microscopy

To visualize colonies using scanning electron microscopy (SEM), agar disks (⌀ 8 mm) containing colonies grown on 80%MYM/20%LPMA plates were isolated using a hole puncher. The samples were prepared as described in the protocol of Yagüe *et al.* [25]. Cells were mounted on an SEM stub and sputter-coated with a 10 nm layer of platinum–palladium. The specimens were observed in a JSM-7600F field emission scanning electron microscope (JEOL) at 5.0 kV.

#### 2.3.4. Transmission electron microscopy

For transmission electron microscopy (TEM), colonies grown on MYM/LPMA (80:20) plates were isolated. Samples were prepared as described by Yagüe *et al.* [25]. The sliced samples were placed on copper TEM support grids and observed using a JEM-1400 Plus Electron Microscope (JEOL) at 80 kV.

#### 2.3.5. Cryo-electron microscopy

For cell wall thickness measurements, sacculi were isolated and analysed using cryo-electron tomography. 32 h old liquid cultures grown in TSBS were resuspended in cold 0.1 M TrisHCl (pH 7.0), subsequently boiled in 4% sodium dodecyl sulfate (SDS) for 30 min, and washed with MilliQ. Samples were then enzymatically treated with DNase, RNase and trypsin. After overnight incubation, the sample was again boiled for 30 min in 4% SDS and washed. Colloidal gold beads with a size of 10 nm were added to the samples in a 1:25 ratio, after which 3 µl was applied to a glow-discharged R2/2 200 mesh holey carbon EM grid (Quantifoil). The samples were vitrified in liquid ethane using an EM GP automated freeze-plunger (Leica) and observed using a single tilt specimen holder inside a 120 kV Talos L120C TEM (ThermoFisher) with a Lab6 electron source and Ceta detector at the Netherlands Centre for Nanoscopy (NeCEN). Cell wall thickness was measured as described [26].

### 2.4. Western blot

For the detection of 6xHis-tagged DivIVA, L-form strains were grown in LPB in a shaker for 2 days at 30°C, 100 RPM till OD = 0.4. After centrifugation, the cells were then harvested by resuspension in 500 μl lysate buffer supplemented with EDTA-free protease inhibitor and by vortexing for 1 min. Total protein concentration was measured using the Bradford protein assay. Samples were then diluted in lysate buffer supplemented with EDTA-free protease inhibitor to a concentration of 0.67 μg μl^−1^. The samples were mixed in a 5:1 ratio with loading buffer and boiled for 5 min at 95°C. 10 μg of total protein was separated on SDS-polyacrylamide gels at 150 V for 80 min. Proteins were transferred electrophoretically to 0.2 µm nitrocellulose membrane. The membrane was then blocked with blocking solution (5% milk in PBS buffer), and incubated with 1:5000 diluted anti-His antiserum (Thermo Fisher, catalogue # MA1-21315) for overnight at 4°C. Subsequently, the membrane was washed three times in PBS buffer, after which the membrane was incubated with 1:5000 diluted anti-mouse IgG (whole molecule)-alkaline phosphatase antibody (Merk, A3562), and washed three times in TBST. The blot was then incubated with AP buffer containing NBT and BCIP to visualize the bands for His-tag signals.

### 2.5. ImageJ analysis

To measure colony size, shape and area, 30 colonies were picked per strain by randomly generating *x*- and *y*-coordinates and selecting the nearest free-lying colony to that point. Images were then measured with ImageJ version 1.53 k by using a 5 mm reference image as a scale, after which the MeasureColony.js macro was run [27]. Alternatively, the colony was selected with the magic wand tool or manually traced with the freehand selection tool. ‘Area’ and ‘Shape descriptor’ were automatically measured by ImageJ. Data was analysed with R version 4.4.0, using the packages ‘car’ and ‘ggpubr’.

### 2.6. Protein structure prediction, analysis and visualization

Predictions of protein structures were created with AlphaFold3 [28], installed locally on High Performance Computing (HPC) facility ALICE. Structures were predicted using 100 randomly generated seeds, from which the highest confidence scoring models were selected by the chain pair predicted aligned error (PAE) values. Visualization was performed using PyMOL to extract interacting residues.

### 2.7. Sequence motif analysis

To identify conserved motifs, the Multiple Expectation Maximization for Motif Elicitation (MEME) software suite [29] was used with the MEME algorithm (version 5.3.2) [30] to discover novel, ungapped motifs without indels. Default settings were used with the anr (any number of repeats) modus, and the maximum number of motifs was set to 8. From the phylum Actinomycetota, DivIVA proteins from the following species were used: Order Mycobacteriales: *Corynebacterium camporealensis, C. glutamicum, M. smegmatis*, *Mycobacterium tuberculosis, M. xenopi, Rhodococcus erythropolis*; Order Micrococcales: *Micrococcus luteus*; Order Streptomycetales: *K. viridifaciens, Streptomyces coelicolor, S. hygroscopicus*. From the phylum Bacillota, DivIVA proteins from the following species were used: Order Bacillales: *Bacillus atrophaeus, B. pseudomycoides, B. subtilis*; Order Eubacteriales: *Clostridioides difficile*; Order Lactobacillales: *Enterococcus faecalis, E. villorum, Streptococcus pneumoniae, S. pyogenes, S. suis*; Order Paenibacillales: *Paenibacillus mucilaginosus*.

## 3. Results

### 3.1. Construction and functional analysis of DivIVA variants

**Figure 1.**
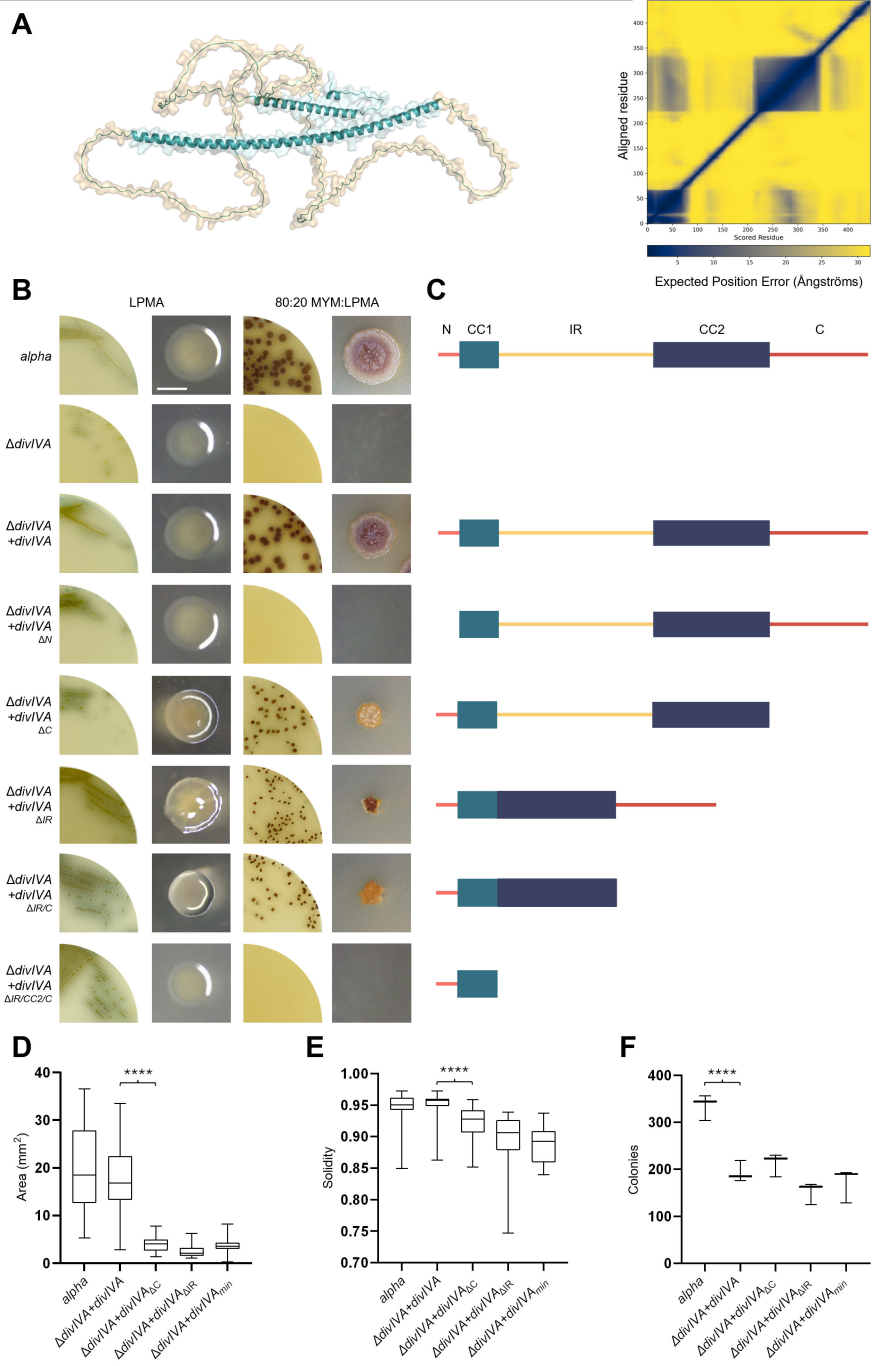
Phenotypic analysis of *K. viridifaciens* Δ*divIVA* expressing *divIVA* variants. (A) Cartoon and surface representation of DivIVA of *K. viridifaciens* made using AF3. Structured domains are represented in blue, and unstructured domains are depicted in light brown. The PAE plot is shown on the right. Low confidence values are yellow, high values are blue. pLDDT values—per-residue measures of local confidence—can be found in electronic supplementary material, figure S1. (B) L-form strains expressing different *divIVA* alleles were tested for growth as L-forms on LPMA medium (left two images) and for the ability to revert to walled growth on non-osmoprotective plates (right two images). Scale bar = 2 mm. (C) Schematic overviews showing the architecture of the DivIVA variants from panel B, relative to the wild-type protein (top), which contains four domains: an N-terminal domain (M1-N65), which we have subdivided into a membrane-binding structure (N) (M1-D23) (light red line) and a coiled-coil region (CC1) (E24-N65) (light blue box), an intercoil region (IR) (M66-G224) (yellow line), a larger coiled-coil domain (CC2) (D225-P345) (dark blue box), and a C-terminal domain (C) (P346-N445) (dark red line). (D) Quantification of the average colony area for strains expressing DivIVA variants (*n* = 30). (E) Quantification of colony solidity for strains expressing DivIVA variants (*n* = 30). Solidity was calculated by dividing the colony area by the convex area. (F) The number of reverted colonies per strain per reversion plate (*n* = 3).

DivIVA is essential for polar growth of Actinomycetota, but it can be deleted in *Kitasatospora viridifaciens* L-forms, as these replicate via biophysical processes instead of hyphal extension [19]. Strain Δ*divIVA* cannot revert to mycelial growth, except if it is complemented by a functional copy of *divIVA* [19]. In streptomycetes, DivIVA consists of four domains: an N-terminal domain, which comprises a coiled-coil segment with a membrane-targeting structure at its N-terminus, followed by an intercoil region, a second coiled-coil and a C-terminal domain [5]. Alphafold3 (AF3) models predict that the intercoil domain and C-terminal domain of *K. viridifaciens* DivIVA are unstructured, as shown by the PAE plot (figure 1A). To determine which of these four domains is essential for walled growth, we engineered *divIVA* alleles encoding variants lacking one or more of these domains (figure 1B,C) and expressed them in the *divIVA* null mutant. All generated mutant strains expressing one of the DivIVA variants retained their capacity to grow as L-forms (figure 1B). However, transferring the strains to reversion plates [22] revealed major differences in terms of their morphology and their efficiency in reverting to a walled state. As expected, expression of wild-type DivIVA enabled the Δ*divIVA* mutant to revert (figure 1B). Reversion could not be achieved when the membrane binding domain was absent (figure 1B), consistent with previous studies [4,31]. Notably, reversion occurred not only when either the intercoil region or the C-terminus of DivIVA were removed (figure 1B), but also when both domains were deleted (figure 1B). Deletion of one or both unstructured domains resulted in significantly smaller and asymmetrical colonies as compared with strains expressing the wild-type protein, with notable differences in roundness calculated by solidity (pairwise *t*‐test with Holm multiple testing correction, *p* < 0.0001) (figure 1D,E). Furthermore, we detected no significant difference in the reversion efficiency among the complemented strains. Still, all these strains that expressed *divIVA in trans* reverted significantly less efficiently than *alpha* (one-way ANOVA, *p* < 0.0001) (figure 1F). When additionally the second coiled-coil domain of DivIVA was deleted from the version already lacking the intercoil region and the C-terminal part, the ability to complement Δ*divIVA* and support reversion was lost (figure 1B). This indicated that DivIVA_ΔIR/C_ (hereinafter referred to as DivIVA_min_) represents a minimized DivIVA protein capable of supporting walled growth in *K. viridifaciens*.

### 3.2. Deletion of the unstructured domains affects mycelial morphology

**Figure 2.**
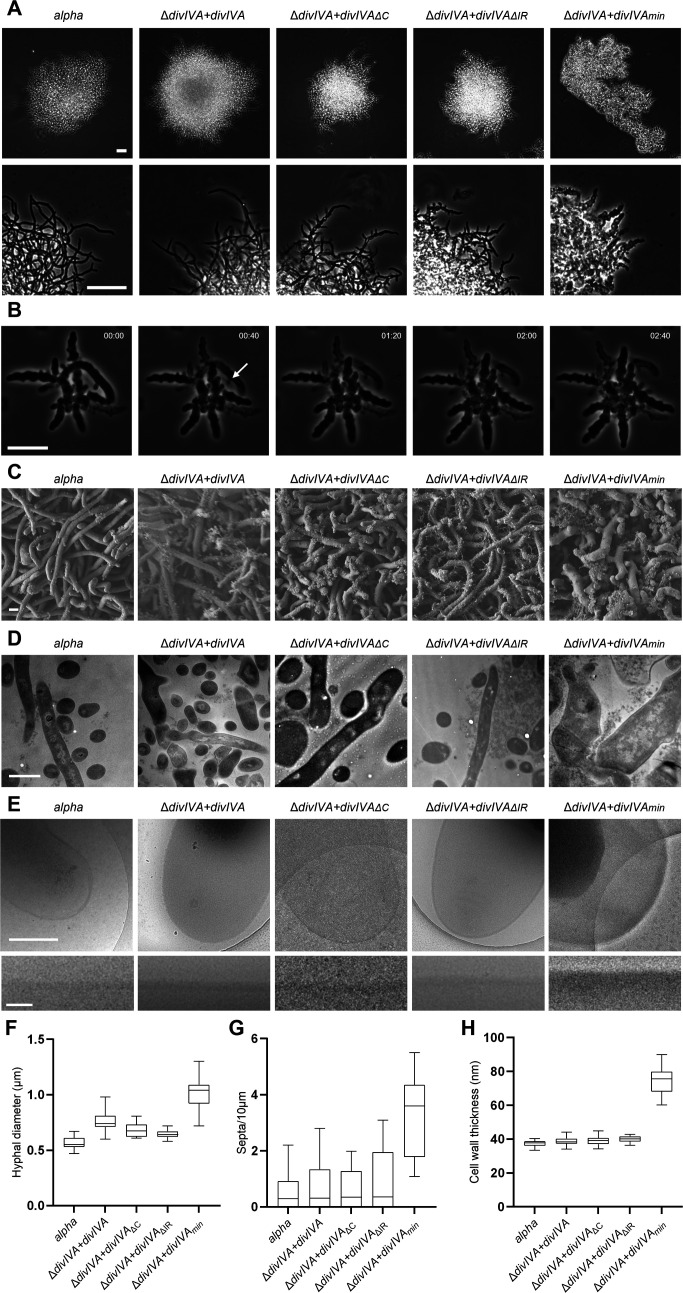
Morphological characterization of strains expressing distinct DivIVA variants. (A) Phase-contrast light microscopy pictures of mycelia of Δ*divIVA* transformants expressing different *divIVA* variants grown overnight in TSBS without shaking. Scale bar = 20 µm. (B) Time-lapse sequence showing bursting of a hypha (white arrow) of *ΔdivIVA + divIVA_min_* observed in a microfluidics chamber. Mycelia were grown in TSBS and loaded into a CellASIC ONIX2 system. Time is in h:min. Scale bar = 10 µm. (C) Scanning electron micrographs of strains *Alpha* and *ΔdivIVA + divIVA*, *ΔdivIVA + divIVA_ΔC_*, *ΔdivIVA + divIVA_ΔIR_* and *ΔdivIVA + divIVA_min_* grown as mycelium on MYM:LPMA (4:1). Scale bar = 1 µm. (D) Transmission electron micrographs of thin-sections of mycelia of the five revertant strains grown on MYM:LPMA (4:1). 70 nm slices were made and placed on copper TEM support grids. Scale bar = 1 µm. (E) Cryo-electron micrographs of hyphal tips of five revertant strains grown in TSBS. Isolated sacculi were vitrified and observed inside a 120 kV Talos L120C TEM. The bottom panel depicted the corresponding straightened cell wall at the apical region of each strain. Scale bar top panel = 500 nm. Scale bar bottom panel = 100 nm. (F) Hyphal diameter of the five revertant strains calculated from SEM data (*n* = 15). (G) The number of septa measured for mycelia with a length >4 µm calculated from TEM data (*n* = 12). (H) The average subapical cell wall thickness measured for each strain from cryo-electron micrographs (*n* = 15). Cell wall thickness was calculated by averaging pixel values (in arbitrary units) along the straightened cell wall and normalized against the background pixel values.

To further study the effect of deleting the unstructured domains of DivIVA on growth and morphogenesis, reverted colonies were inoculated in TSBS medium and subsequently transferred to TSBS medium in non-shaking flasks. As expected, the morphology of the strain expressing the ectopic copy of native *divIVA* was identical to that of *alpha* (figure 2A). Deleting either the intercoil or the C-terminal region impacted branching dynamics, as mycelia of Δ*divIVA + divIVA*_Δ_*_IR_* and Δ*divIVA + divIVA*_ΔC_ were irregular and more highly branched with branches closer to the tip compared with Δ*divIVA + divIVA* (figure 2A). Δ*divIVA + divIVA_min_* showed a more extreme phenotype characterized by short and wide hyphae of highly irregular shape and cell dimension, and more compact mycelial clumps than in the single domain deletions (figure 2A). Live-cell time-lapse imaging showed that, in contrast to *ΔdivIVA + divIVA* (electronic supplementary material, video S1), hyphae of Δ*divIVA + divIVA_min_* showed frequent localized bursting of hyphae at the tips (figure 2B, electronic supplementary material, videos S2, S3). Scanning electron microscopy revealed that hyphae of Δ*divIVA + divIVA_min_* exhibited an abnormal, widened morphology, characterized by kinked shapes and short outgrowths (figure 2C). Measurements of hyphal thickness in scanning electron micrographs revealed that the hyphae were, on average, 1.7× wider in Δ*divIVA + divIVA_min_* compared with Δ*divIVA + divIVA* (figure 2F). For counting the number of septa per strain, our initial goal was to use membrane staining in combination with fluorescence microscopy. However, the mycelia of Δ*divIVA + divIVAmin* grown in liquid cultures were so dense that this prevented differentiation between hyphae when performing fluorescence microscopy (data not shown). Therefore, we made thin-cutting sections of hyphae from colonies, which were observed using transmission electron microscopy. This revealed an average 4.9× increase in the number of septa in *ΔdivIVA + divIVA_min_* compared with *ΔdivIVA + divIVA* (*n* = 20) (figure 2D,G). Using cryo-electron tomography, we also found an average 1.9× increase in subapical cell wall thickness between *ΔdivIVA + divIVA* and *ΔdivIVA + divIVA_min_* (figure 2E,H). In conclusion, these data show that deletions of the unstructured domains, namely the intercoil region and C-terminus, have pleiotropic effects on the morphology, affecting branching frequency, and that deleting both these domains to make the *divIVA_min_* allele leads to a very strong phenotype, with increasing cross-wall formation, highly irregular hyphal diameter, increased cell-wall thickness and frequent lytic events.

### 3.3. Chimeric DivIVA functionality in *K. viridifaciens* is limited to Actinomycetota

**Figure 3.**
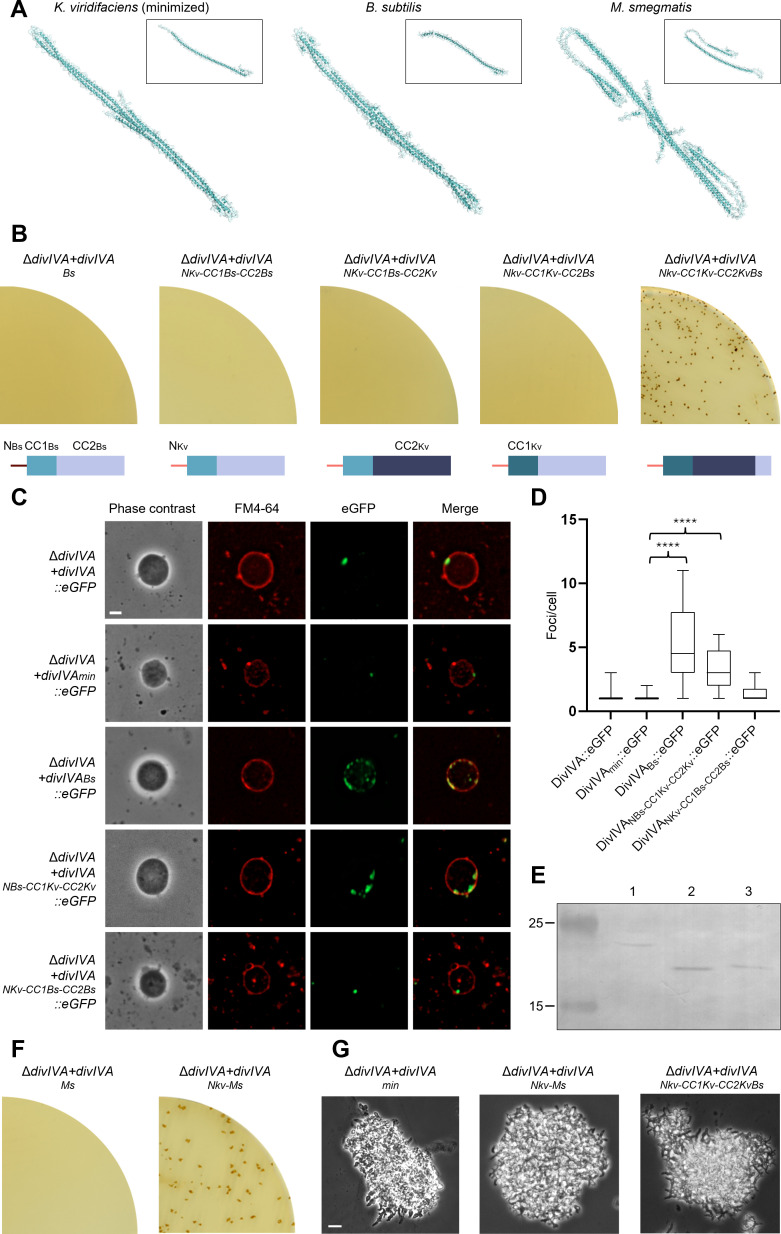
Functionality of chimeric DivIVA in *K. viridifaciens* is limited to Actinomycetota. (A) Cartoon and surface representation of monomers and predicted tetramers of minimized DivIVA of *K. viridifaciens*, and DivIVA from *B. subtilis* and *M. smegmatis*, made using AF3. PAE plots are shown in electronic supplementary material, figure S3. (B) L-form strains expressing different chimeric *divIVA* alleles of *B. subtilis* and *K. viridifaciens* were tested for the ability to revert to walled growth on non-osmoprotective plates. Schematic overviews show the architecture of the DivIVA variants relative to the wild-type *B. subtilis* protein, which contains a membrane-binding domain (N) (M1-D21) (reddish-brown line) and a coiled-coil region, which we have divided into smaller coiled-coil (CC1) (E22-E64) (light blue box) and a larger coiled-coil (CC2) (E65-E164) (lila box), relative to the coiled-coil regions in *K. viridifaciens* DivIVA. (C) eGFP was fused to the C-terminus of various DivIVA proteins to check for self-association behaviour. Image stacks along the Z-axis (spacing 270 nm) were collected and deconvolved, and orthogonal projections were made from the four focal planes around the centre of the cells. L-form cells were placed on 1% agarose in P-buffer with 0.5 μg ml^−1^ FM4-64 for membrane labelling. Scale bar = 2 µm. (D) The number of DivIVA::eGFP foci was counted per cell throughout the orthogonal projections (*n* = 20). (E) Western blot of DivIVA_min_-his [1], DivIVA_Bs_-his [2] and DivIVA_NKv-CC1Bs-CC2Bs_-his [3]. L-form strains were grown in LPB in a shaker for 2 days to OD = 0.4. Equal amounts of total cell protein were loaded in each well. The positions of molecular weight size markers (kDa) are indicated. (F) L-form strains expressing different chimeric *divIVA* alleles of *M. smegmatis* were tested for the ability to revert to walled growth on non-osmoprotective plates. (G) Light microscopy pictures of mycelia of Δ*divIVA + DivIVA_min_*, Δ*divIVA + divIVANkv_Nkv-Ms_* and Δ*divIVA + divIVANkv_Nkv-CC1Kv-CC2KvBs_* grown overnight in TSBS without shaking. Scale bar = 10 µm.

AF3 modelling predicts that deletion of the intercoil region and the C-terminal domain of DivIVA results in a fusion of the N-terminal domain and the second coiled-coil (figure 3A). Furthermore, predicted structures of DivIVA_min_ revealed that its self-association results from a C-terminal motif that enables antiparallel dimer–dimer binding, resulting in a tetramer (figure 3A). We also attempted to model the tetramerization of the full-length DivIVA protein. However, the models showed low confidence, likely due to the presence of the two unstructured domains, which prevented a conclusive interpretation. Although DivIVA_min_ has structural similarities with *B. subtilis* DivIVA (figure 3A), there are sequence differences in motifs found in the first and second coiled-coil domains between Actinomycetota and Bacillota (electronic supplementary material, table S4). These differences might reflect the distinct growth modes: *B. subtilis* elongates through the lateral insertion of cell wall material [10], whereas *K. viridifaciens* grows by polar extension [32].

Complementation experiments revealed that *B. subtilis* DivIVA could not restore walled growth in the *ΔdivIVA* mutant of *K. viridifaciens* (figure 3B). We initially hypothesized that this failure was due to improper localization. Indeed, localization studies with C-terminal eGFP fusions revealed that *B. subtilis* DivIVA formed multiple foci rather than a single focus or a few distinct foci (figure 3C). Replacing its membrane-binding domain with that of DivIVA_min_ significantly improved localization, resulting in one or a few foci similar to DivIVA_min_ (figure 3C,D). However, this improvement remained insufficient to restore functionality (figure 3B). Furthermore, Western blot analysis showed that DivIVA levels were comparable between the *ΔdivIVA + divIVA*_*min*_*-his*, *ΔdivIVA + divIVA*_*Bs*_*-his* and *ΔdivIVA + divIVANKv*_*NKv-CC1Bs-CC2Bs*_*-his* strains (figure 3D). Replacing CC1 or CC2 in DivIVA_min_ with those from *B. subtilis* likewise failed to support reversion (figure 3B). However, functionality was maintained when the last 26 amino acids of DivIVA_min_ (T161-P186) were replaced with the corresponding residues from *B. subtilis* (Q162-E187) (figure 3B). This likely reflects the restricted role of (a part of) these residues as an unstructured tail in the antiparallel binding structure downstream of the second coiled-coil region (electronic supplementary material, figure S4).

By contrast, while native DivIVA from the polar-growing *M. smegmatis* could not restore reversion, a chimeric *M. smegmatis* DivIVA protein in which the membrane binding domain (M1-N24) was replaced with that of *K. viridifaciens* (M1-D23) could (figure 3B), resulting in a morphology similar to that of DivIVA_min_ (figure 3C). Notably, Alphafold3 (AF3) models predict that DivIVA from *M. smegmatis* has an unstructured region starting at the same position as in *K. viridifaciens* DivIVA (figure 3A, electronic supplementary material, figure S3). Together, these results suggest that, despite structural similarities, DivIVA proteins have diverged functionally in response to different growth modes. This highlights the importance of specific amino acid sequence elements for proper localization, differentiation and cellular function.

## 4. Discussion

DivIVA is a coiled-coil protein widely conserved in both Actinomycetota and Bacillota. In Actinomycetota, such as *K. viridifaciens* and *M. smegmatis*, DivIVA plays an essential role in polar growth [5,6,8], whereas in Bacillota, such as *B. subtilis*, DivIVA plays a non-essential role in cell division [10]. So far, it has been impossible to functionally replace *divIVA* in Streptomycetaceae with those of other taxa. To analyse DivIVA and polar growth, we here exploited an L-form *divIVA* deletion strain derived from *K. viridifaciens*, as this strain replicates via biophysical processes instead of hyphal extension [19]. These cells can only reinitiate walled growth in the presence of a functional form of DivIVA. Using this platform, we have investigated the minimal structural requirements of DivIVA to facilitate walled growth in *K. viridifaciens*. After deleting the intercoil region and the C-terminal domain, both of which we have shown to be unstructured domains, we have built a minimized DivIVA protein consisting of the N-terminal domain and the second coiled-coil.

DivIVA forms a multimeric complex in streptomycetes [4]. Our AlphaFold3 models predict that DivIVA_min_ assembles into a tetramer, similar to DivIVA of *B. subtilis* [12]. When we deleted the second coiled-coil domain in DivIVA_min_, this prevented reversion to a walled state in *ΔdivIVA*. In *Streptomyces coelicolor*, deleting the second coiled-coil of DivIVA disrupted oligomerization [4]. In *Corynebacterium glutamicum*, the N-terminal domain and two coiled-coil regions have already been shown to be essential for DivIVA functioning [31]. In *B. subtilis*, the C-terminal coiled-coil region, which correlates to the second coiled-coil in *K. viridifaciens*, is involved in oligomerization [12]. In *Deinococcus radiodurans*, purified DivIVA showed bundles under TEM, whereas the variant consisting of the N-terminus and the first coiled-coil did not [33]. Furthermore, the C-terminal region seemed to be crucial for the structural and functional integrity of DivIVA [33]. Altogether, this shows that the multimerization behaviour of DivIVA is conserved among bacterial taxa.

Although our minimal DivIVA protein is structurally similar to that of *B. subtilis*, production of *B. subtilis* DivIVA in Δ*divIVA* could not facilitate reversion to a walled state. Substituting the membrane-binding structure of the N-terminal domain with that of *K. viridifaciens* resulted in a comparable number of foci to DivIVAmin, yet still failed to support reversion. This may reflect differences in membrane affinity, as simulations have predicted distinct membrane interactions for DivIVA from *S. coelicolor* and *B. subtilis* [9]. When we replaced CC1 or CC2 in DivIVAmin with that of *B. subtilis*, this also did not facilitate reversion (figure 3B). Notably, Actinomycetota possess different amino acid motifs in their coiled-coil domains compared with Bacillota, suggesting functional divergence. Previous studies have shown that DivIVA from *Mycobacterium tuberculosis* and *Streptomyces coelicolor* can compensate for DivIVA depletion in *C. glutamicum* and restore polar growth, whereas *B. subtilis* DivIVA cannot [7]. Interestingly, either coiled-coil domain could be swapped with the corresponding domain from *B. subtilis* without loss of function, provided the N-terminal domain of *C. glutamicum* DivIVA remained intact [31]. In our experiments, replacing only the last 26 amino acids of DivIVAmin with those from *B. subtilis* enabled reversion, likely reflecting a limited role of a part of these residues as an unstructured tail. By contrast, when we substituted the N-terminal domain with that of *K. viridifaciens*, DivIVA from *M. smegmatis* facilitated reversion. Together, these results indicate that polar growth in Streptomycetaceae depends not only on overall structural features but also critically on specific amino acid sequences.

Deleting either the intercoil or the C-terminal region impacted branching dynamics, resulting in irregularly shaped strains with increased branching and shorter distances between the tips and new branches. Deletion of both domains caused more severe morphological defects, including irregular hyphal diameter, reduced septation, thinner cell walls and frequent tip bursting. These defects may result from the loss of interactions with key partner proteins. DivIVA is known to interact with several cytoskeletal components, including Scy and FilP, forming a dynamic complex at the hyphal tip that coordinates the insertion of new cell wall material [34–36]. It also interacts with CslA, a cellulose synthase-like protein involved in β-glucan biosynthesis at growing tips [37–39]. In our study, deletion of the C-terminal region likely disrupted DivIVA phosphorylation, as five *in vivo* phosphorylation sites have been identified in this region in *Streptomyces coelicolor* [40]. One of the kinases involved is AfsK, a serine/threonine protein kinase [41], while the phosphatase SppA counteracts this modification [24]. Future work should clarify the roles of the unstructured regions and determine which interaction partners are lost upon their deletion. Identifying these partners in *Kitasatospora viridifaciens* would be the first step, followed by mapping their binding domains within DivIVA, which could then be validated using two-hybrid screening. In conclusion, our findings demonstrate that a minimized DivIVA protein is sufficient to initiate polar growth in Streptomycetaceae, but fails to preserve the complex mycelial architecture, underscoring the essential role of the full-length protein in maintaining filamentous growth. This highlights a crucial, yet undefined role for the unstructured regions in the morphogenesis of Streptomycetaceae.

## Data Availability

Data are available on Figshare [42].
